# MIlitary Combat Mental Health Framework

**DOI:** 10.1136/bmjmilitary-2020-001439

**Published:** 2020-04-22

**Authors:** Martin Bricknell

**Affiliations:** Conflict and Health Research Group, King's College London – Strand Campus, London, UK

**Keywords:** mental health, occupational & industrial medicine, psychiatry

## Abstract

This paper describes a framework for understanding military combat mental health based on the possible mental ill-health consequences of exposure to ‘potential trauma events’ for members of the armed forces and after their military service as veterans. It uses a life course approach that maps an individual’s mental well-being against four ‘states’: fit, reacting, injured and ill. It then considers five categories of factors that influence the risk of mental illness from this exposure based on research evidence; prejoining vulnerability, resilience, precipitating, treatment and recovery. This framework offers a structure to debate current knowledge, inform policy and therapeutic interventions, provide education and to guide future research into the subject.

**Key messages**

- Exposure to potential trauma events (PTEs) may cause mental ill-health in armed forces personal and veterans at a later period in their life course.
- Mental health in this population can be considered to lie on a continuum from fit (healthy), reacting, injured and ill.
- Risk factors for mental ill-health from a PTE can be grouped into prejoining vulnerability, resilience, precipitating, treatment and recovery.
- The Military Combat Mental Health Framework may provide a useful conceptual model to plan mental and social health services, conduct research and collaborate across service providers.

## Introduction

Armed forces and veteran populations may have exposures to combat that cause a severe impact on their physical, mental or social health. The primary UK long-term cohort study of a sample of UK military personnel and veterans from the wars in Iraq and Afghanistan has shown a prevalence of symptoms of 21.9% for common mental disorders, 10.0% for alcohol misuse and 6.2% for probable post-traumatic stress disorder (PTSD).^1^ PTSD was the most common clinical keyword in the global military medical literature between 1988 and 2017.^2^ While there is a substantial body of research on military service and mental health, there are very few models that integrate this evidence to inform policy and practice. The purpose of this paper is to offer an explanatory framework to support further debate on risk, treatment and social interventions for combat-related mental ill-health in armed forces personnel and veterans. However, it should be noted exposure to combat is not the primary cause of mental ill-health in this population and that the overall incidence of mental ill-health is less than an age-matched group of the overall UK population.^3^

## The Military Combat Mental Health Framework

**Figure 1:**
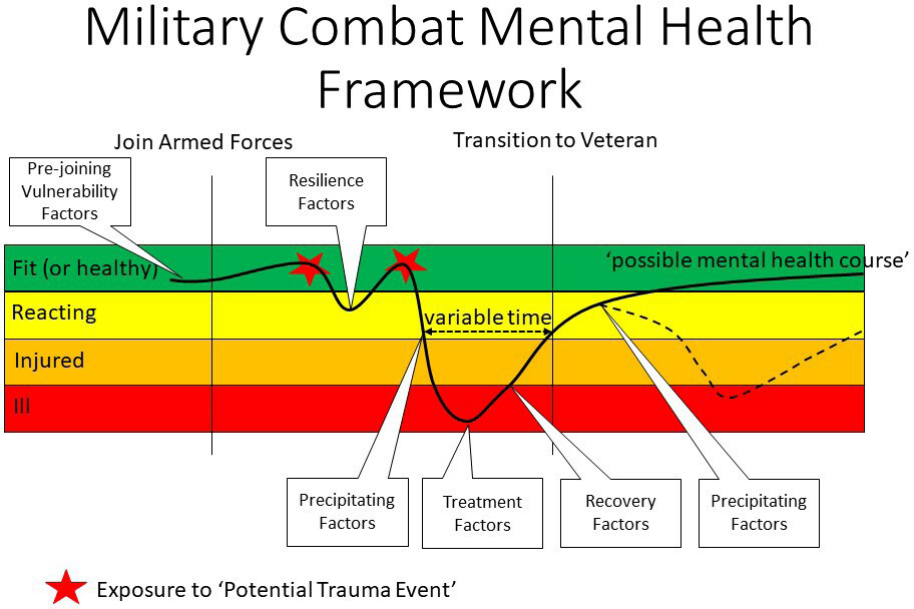
Military Combat Mental Health Framework.

In October 2019, the North Atlantic Treaty Organisation published Standardisation Agreements for military medical services on deployment mental health.^4 5^ While not all mental illness resulting from combat exposure is PTSD, a meta-analysis of risk factors for combat-related PTSD^6^ and a comprehensive review of military-related PTSD are valuable summaries of the research evidence.^7^ These papers are the basis for the Military Combat Mental Health Framework shown in Figure 1. Exposure to one or more ‘potential trauma events’ (PTE) is essential for causation. A PTE is an event occurring as a result of combat that involved actual or threatened death or serious injury, or threat to the physical integrity of self or others. The framework extends from before military service through to postmilitary life as the mental health consequences of exposure to combat-related stress may be delayed and first present in veterans.^8^

An individual’s mental well-being may fluctuate on a continuum between ‘Fit’, ‘Reacting’, ‘Injured’ and ‘Ill’ defined by function within their communities. This ‘community’ context is important because it is family, friends and coworkers who may notice the consequences of mental ill-health before the individual recognises or accepts their condition. The framework uses the following definitions:

### Fit (or healthy)

Individuals in this zone have a state of balanced ‘well-being’ across all domains of health and are functional for their role within work, personal life and social environment (communities). This extrapolates the concepts behind physical fitness (trained optimal performance) into mental and social fitness.

### Reacting

Individuals within this zone are experiencing mild, transient stress reactions as a result of their combat-related experiences. These are a common human reaction to the realities of combat and are recognisable if explained to third party.

### Injured

This state includes more severe, persistent symptoms that result in abnormalities in an individual’s behaviour and are not getting better. A third party may notice this change because of changes in the individual’s functioning within their communities, but their condition would not meet the criteria for a formal clinical diagnosis.

### Ill

Illness occurs when an individual’s combat experience has a significant impact on their function within their communities, indicating a severe disorder that can be clinically diagnosed, such as major depression, anxiety or PTSD.

The framework shows a hypothetical trajectory of an individual’s mental health course through their life after joining the armed forces. The initial trajectory assumes improved mental fitness compared with entry to the armed forces as military training is designed to increase their physical fitness and provide psychological preparation for their military role. This experience and their social community help to develop personal *resilience factors*. Until the first PTE, military personnel have no combat-related mental ill-health and have the potential to be mentally Fit. The first PTE may result in a stress reaction (reacting), but they may be able to return to fitness because of their resilience factors. They could then experience a second PTE, which may compound the effects of the first PTE. After a variable duration, this exposure may cause a progressive deterioration of their mental health becoming injured or ill as a result of *precipitating factors*. Successful therapeutic interventions to improve from illness will depend on *treatment factors*, though long-term mental health will depend on *recovery factors*. Unfortunately, a permanent ‘cure’ may not be possible, and individuals may deteriorate again as a result of the re-emergence of precipitating factors. This is shown as happening in the individual’s life course after military service as a veteran.

## Risk and protective factors

There is strong epidemiological evidence that an individual’s risk of military combat-related mental illness can be associated with a range of factors that accrue during their life course. These are categorised into the following groups with examples in each.

### Prejoining vulnerability factors

Prejoining vulnerability factors are adverse life experiences that occur prior to military service. The existence of these is fixed but their impact will vary according to subsequent life events. They are common in the population that seek to join the armed forces and include: childhood adversity and childhood antisocial behaviour; low educational attainment (which has an influence in choice of military employment, the likelihood of being employed in a combat role and socioeconomic status as a veteran); and pre-service mental ill-health (though most militaries exclude applicants with a previous mental health diagnosis). While these risk factors are not suitable as screening criteria for exclusion from military service, there may be value in identifying them in those that seek help after a PTE in order to inform clinical interventions.

### Resilience factors

Resilience factors are those, post-entry training, organisational and personal interventions that reduce (or the absence of which increase) the risk that an individual may experience mental illness as the result of exposure to a PTE. These may include: not being deployed as a reservist; absence of alcohol or substance abuse; being a non-smoker; absence of previous mental ill-health; not in a combat role (though this may be solely due to reduction in risk of exposure to a PTE); being in a personal relationship; unit cohesion and leadership; rest and recuperation during operational deployment; post-tour third location decompression; higher rank; recipient of Trauma Risk Management support (TRiM); deployment length less than 6 months; and lower total number of deployments. Many of these factors are amenable to organisational interventions at the group level.

### Precipitating factors

A severe PTE may result in an acute stress disorder (immediate onset of debilitating mental illness). However, there may also be a time lag between exposure, the onset of mental ill-health and seeking help. Precipitating factors are those that trigger a deterioration to the extent that the individual becomes mentally injured or ill. These may include: concurrent physical illness or injury, severity of PTE, poor sleep, physically aggressive behaviour and concurrent alcohol misuse. There may be differences between the factors that precipitate combat-related mental ill-health while in military service and those factors that apply to veterans who seek help. Thus, a separate ‘Precipitating Factors’ box is shown after transition to veteran.

### Treatment factors

Although there are clearly established clinical modalities for the treatment of combat mental illness, there is variation in the clinical outcomes of these therapies for individual patients. Some of this variation can be explained by adverse clinical and non-clinical treatment factors such as: presence of non-PTE concurrent mental illness; poor outcomes from concurrent physical illness; chronic pain; older age; duration of functional impairment prior to seeking treatment; severity of functional impairment; perception of stigma to seeking help; and lack of an internal locus of control.

### Recovery factors

The final group of factors is labelled recovery factors. These are the psychosocial factors that facilitate recovery from the injured through to the fit zones and mitigate against the precipitating factors causing a recurrence of a deterioration in mental well-being for the individual. All military personnel will transition to veteran at some point in their life and so these factors apply both during and after military service. Positive recovery factors include: not smoking; no substance or alcohol abuse; no risk-taking behaviour; no homelessness; no criminal activity; post-traumatic growth; remaining in military service; being in employment; having a personal relationship; and no decline in cognition in later age. Social networks play an important role in personal resilience with an increase in common mental disorders and PTSD symptoms in service leavers after they have left their social network in the military. This can extend to non-healthcare social interventions such as adventurous activities, surfing and ‘animal therapies’.

## Interpretation of the framework

This framework adds to those that already exist by illustrating mental health across the life course from before military service to becoming a veteran. It categorises risk factors to inform individual, organisational and therapeutic interventions to mitigate and manage the risks from exposure to a PTE on an individual’s mental health. The actual experience of mental well-being or ill-health for an individual may not exactly follow the trajectory shown. The framework de-emphasises the notion of a permanent ‘cure’ for mental ill-health and recognises the inter-relationships between physical, mental and social domains of health. This reinforces the importance of the social domain of health and the need to consider ‘social illness’ and ‘social interventions’ alongside mental illness and mental interventions. It also highlights the potential for community and social interventions during the ‘Reacting’ and ‘Injured’ stage when interventions by clinical professionals may not be required or appropriate.

The framework has created categories for the various factors that influence the risk of combat-related mental ill-health. These categories may need further refinement to develop the best descriptors for each factor and to identify those factors with positive attributes alongside negative risk factors. The prejoining vulnerability factors are not sufficiently specific or sensitive for use as screening criteria to exclude individuals from joining the armed forces. There is also evidence that postdeployment screening for mental ill-health in military personnel was not effective reducing the prevalence of mental disorders in the UK population.^9^ However, this does not remove the need for general awareness of the symptoms and signs of mental ill-health across the military population as demonstrated by the Trauma Risk Management programme and briefings during Third Location Decompression.^10 11^ Knowledge of these factors can also inform the inter-relationship of the responsibilities between the individual, their social circumstances, the armed forces as an employer, the government for statutory services for veterans, and the contribution of charities and the wider the voluntary sector for mitigating mental ill-health in this population.

The time course emphasises the importance of maintaining the observational research cohorts from the deployment to Iraq and Afghanistan in order to observe risk factors of combat-related mental ill-health over the long term. It is also necessary to ensure the replenishment of the cohorts with new entrants to the armed forces so that cohort studies can be rapidly initiated at the beginning of the next combat operation.

Finally, the model could apply to non-military populations with similar exposures such as front-line emergency responders, journalists and humanitarian aid workers. The model does not illustrate the balance of non-PTE exposures (eg, non-combat deployments, separation from personal relationships and work-related stress) as risk factors nor does it accommodate the emerging concept of ‘moral injury’ as a source of mental ill-health in military populations.^12^

## Conclusions

This paper described the Military Combat Mental Health Framework to aid understanding of combat-related mental illness in armed forces personnel and veterans. It is based on a life course approach that maps an individual’s mental well-being against four ‘states’: fit, reacting, injured and ill. The paper described five categories of factors that influence the risk of mental illness from exposure to PTEs: prejoining vulnerability, resilience, precipitating, treatment and recovery. This framework provides a structure to debate current knowledge, inform policy and therapeutic interventions and to guide future research into this subject.
